# Evaluating the Impact of Neurosurgical Rotation Experience in Africa on the Interest and Perception of Medical Students Towards a Career in Neurosurgery: A Continental, Multi-Centre, Cross-Sectional Study

**DOI:** 10.3389/fsurg.2022.766325

**Published:** 2022-02-10

**Authors:** Olaoluwa Ezekiel Dada, Setthasorn Zhi Yang Ooi, George William Bukenya, Yves Jordan Kenfack, Chi Le, Efosa Ohonba, Emmanuel Adeyemo, Kapil Narain, Ahmed K. Awad, Umaru Barrie, Dawin Sichimba, Oloruntoba Ogunfolaji, Lilian Mwende Kitonga, Adaeze Juanita Oriaku, Michael A. Bamimore, Douglas Emeka Okor, Ola Rominiyi

**Affiliations:** ^1^College of Medicine, University of Ibadan, Ibadan, Nigeria; ^2^Cardiff University School of Medicine, Cardiff, United Kingdom; ^3^Case Western Reserve University, Cleveland, OH, United States; ^4^University of Texas Southwestern Medical Center, Dallas, TX, United States; ^5^Vanderbilt School of Medicine, Nashville, TN, United States; ^6^Department of Neurosurgery, Groote Schuur Hospital, University of Cape Town, Cape Town, South Africa; ^7^Nelson R. Mandela School of Medicine, University of KwaZulu-Natal, Durban, South Africa; ^8^Faculty of Medicine, Ain Shams University, Cairo, Egypt; ^9^Michael Chilufya Sata School of Medicine, Copperbelt University, Kitwe, Zambia; ^10^College of Health Sciences, School of Medicine, University of Nairobi, Nairobi, Kenya; ^11^Glan Clwyd Hospital, Bodelwyddan, United Kingdom; ^12^School of Medicine, Philadelphia College of Osteopathic Medicine, Philadelphia, PA, United States; ^13^Department of Neurosurgery, Garki Hospital, Abuja, Nigeria; ^14^Department of Neurosurgery, Sheffield Teaching Hospitals NHS Foundation Trust, Sheffield, United Kingdom; ^15^Department of Neuroscience, University of Sheffield, Sheffield, United Kingdom

**Keywords:** global neurosurgery, clinical rotation, medical students, medical education, training, Africa, interest, perception

## Abstract

**Objective:**

Africa has the second highest neurosurgical workforce deficit globally and many medical students in Africa lack exposure to the field. This study aims to assess the impact of a neurosurgical rotation during medical school in shaping the perception and interest of students toward a career in neurosurgery.

**Study Design:**

Cross-sectional study.

**Methods:**

A Google form e-survey was disseminated to African clinical medical students between February 21st and March 20th, 2021. Data on exposure and length of neurosurgical rotation and perception of, and interest in, neurosurgery were collected. Data was analyzed using descriptive statistics and adjusted logistic regression modeling.

**Results:**

Data was received from 539 students in 30 African countries (30/54, 55.6%). The majority of participants were male and were from Kenya, Nigeria and South Africa. Most students had undertaken a formal neurosurgery rotation, of which the majority reported a rotation length of 4 weeks or less. Students who had more than 4 weeks of neurosurgical exposure were more likely to express a career interest in neurosurgery than those without [odds ratio (OR) = 1.75, *p* < 0.04] and men were more likely to express interest in a neurosurgical career compared to women (OR = 3.22, *p* < 0.001), after adjusting for other factors.

**Conclusion:**

Neurosurgical exposure is a key determinant in shaping the perception and interest of medical students toward a career in neurosurgery. Our findings support the need: i) for a continent-wide, standardized curriculum guide to neurosurgical rotations and ii) to advocate for gender inclusivity in education and policy-making efforts across the African continent.

## Introduction

According to the Lancet Commission for Global Surgery, an estimated five billion people worldwide do not have access to safe, affordable surgical and anesthetic care, with most of these people residing in low- and middle-income countries (LMICs) (1). This health disparity phenomenon is particularly apparent in the neurosurgical landscape seen in Africa.

In recent years, Africa has begun to experience an emerging transformation in the availability of neurosurgical care through an increase in the number of training centers and increased collaboration with international neurosurgical foundations and neurosurgery departments from developed countries (2). However, despite these advances, the continent still has the second highest neurosurgical workforce deficit reported globally (3). This deficit has impacted surgical capacity negatively, with an estimated 1,877,568 surgical case deficit annually (3). Resolving this deficit is critical to ensuring timely access to high-quality neurosurgical care, reducing complication rates, and ultimately improving quality of life.

Interestingly, a recent survey among aspiring African neurosurgeons showed that the vast majority of students have little or no exposure to neurosurgical procedures (4). This highlights the scarcity of avenues for students to gain exposure and nurture their interest in the field. Published literature has shown that an essential long-term strategy in reducing this workforce deficit would include providing medical students with educational and career development opportunities related to the field of neurosurgery (3). This would be a key step in helping students gain an insight into a neurosurgical career and its demands, as well as to identify potential areas of interest.

To date, there has been a paucity of studies appraising the presence of a dedicated neurosurgical rotation in the curriculum delivered in African medical schools. Its impact on the perceptions and interest of medical students toward neurosurgery as a potential career has also yet to be evaluated.

In this prospective cross-sectional study, we aim to assess the role of a defined neurosurgical rotation during medical school and its impact on shaping perceptions and interest toward a neurosurgical career. We also aim to identify the features of a neurosurgical rotation that are associated with positive perception and interest toward a career in neurosurgery. We hypothesize that students with experience of a formal rotation in neurosurgery will have a more positive perception toward a career in neurosurgery.

This is the first study of its kind, addressing a key issue relevant to the whole African continent. Our findings aim to inform the ongoing progressive development of medical curricula, policy, and guidelines in medical schools across Africa.

## Methods

The Strengthening the Reporting of Observational Studies in Epidemiology (STROBE) statement was used to guide this study report (5).

### Study Design

A prospective analytical cross-sectional survey of African medical students was carried out using a self-administered electronic questionnaire. The criteria for the survey eligibility were current medical students studying in Africa and who are in their clinical years (4th to 6th, or higher year of study).

### Data Collection

A 27-item, electronic survey (e-survey) was developed by medical students and a neurosurgery resident using Google Forms (Google, USA). The questionnaire was categorized into four sections: socio-demographic background, neurosurgical experience, perception toward neurosurgical career, and interest in a neurosurgical career. Questions under the perception toward a neurosurgical career category were adapted from Zuckerman et al. (6). The questionnaire included a five-point Likert scale, multiple-choice and free-text questions to improve the granularity of the data collected. All initial questions in the survey required a response to minimize any potential missing data at submission. A pilot survey was distributed to 15 randomly selected clinical medical students in Africa, who were not involved in the conception or design of the study, to seek feedback, improve clarity, and ensure objectivity.

The questionnaire was distributed to clinical medical students in the African continent between February 21st and March 20th, 2021. The questionnaire was available in the English and French language to facilitate broad coverage. The questionnaire in the English language was designed by three authors (OD, EO, OR) and translated to a French version by one author (OD) with the help of a deep learning translator [Traduit avec www.DeepL.com/Translator (version gratuite)]. This translated version of the questionnaire was vetted by a native French-speaking author (YK).

The questionnaires were distributed *via* social media platforms (Twitter, Facebook, WhatsApp, and Telegram), emails, and through executive committee members of medical student associations in some countries. Participation was voluntary, and participants were informed prior to starting the survey that all data collected was non-identifiable and would only be used for the purposes of analysis, distribution and publication. A mandatory selection box consenting to participation and confirming that this was the first time completing this survey was included at the beginning of the survey, ensuring a 100% consent rate. Participants who were unwilling or unable to give consent to the study were excluded. A copy of the final questionnaire can be found in Appendix 1.

### Definition of Terms Used

Formal neurosurgical exposure was defined as having experienced a neurosurgical clinical rotation regardless whether arranged by the medical school or self, informal exposure was defined as experience sought outside of a clinical rotation, and overseas neurosurgical exposure was defined as having experienced a neurosurgical clinical rotation or elective outside Africa. Formal neurosurgical exposure included clinical rotations that may have other specialties taught alongside neurosurgery during the rotation.

### Statistical Analysis

Descriptive statistics were performed for all variables. Depending on normality, the median and interquartile range or mean and 95% confidence interval (CI) were calculated for each domain of perception toward neurosurgery among rotators (defined as students who have undertaken a formal neurosurgical clinical rotation) and non-rotators. Differences in each domain of perception toward neurosurgery were analyzed using Wilcoxon rank sum test for non-parametric variables and the Welch *t*-test for parametric variables. Adjusted logistic regression models were developed to estimate the odds of definite interest in neurosurgery careers. Sequential addition of covariates (age, gender, and geographical location) and likelihood ratio tests of nested models were conducted to identify the model of best fit. The model examined previous formal neurosurgery rotation and length of formal neurosurgery exposure (more than 4 weeks vs. 4 weeks or less). The 4-week cut-off was common practice for the minimum length of clinical rotations in medical schools worldwide (7). The final models were adjusted for gender. The interaction between geographical location and formal rotation experience was detected and included. Statistical significance was accepted at *p* < 0.05. All analyses were conducted on STATA 16.1 (Stata, Version 16.1, StataCorp, USA).

## Results

### Sociodemographic Characteristics

**Figure 1:**
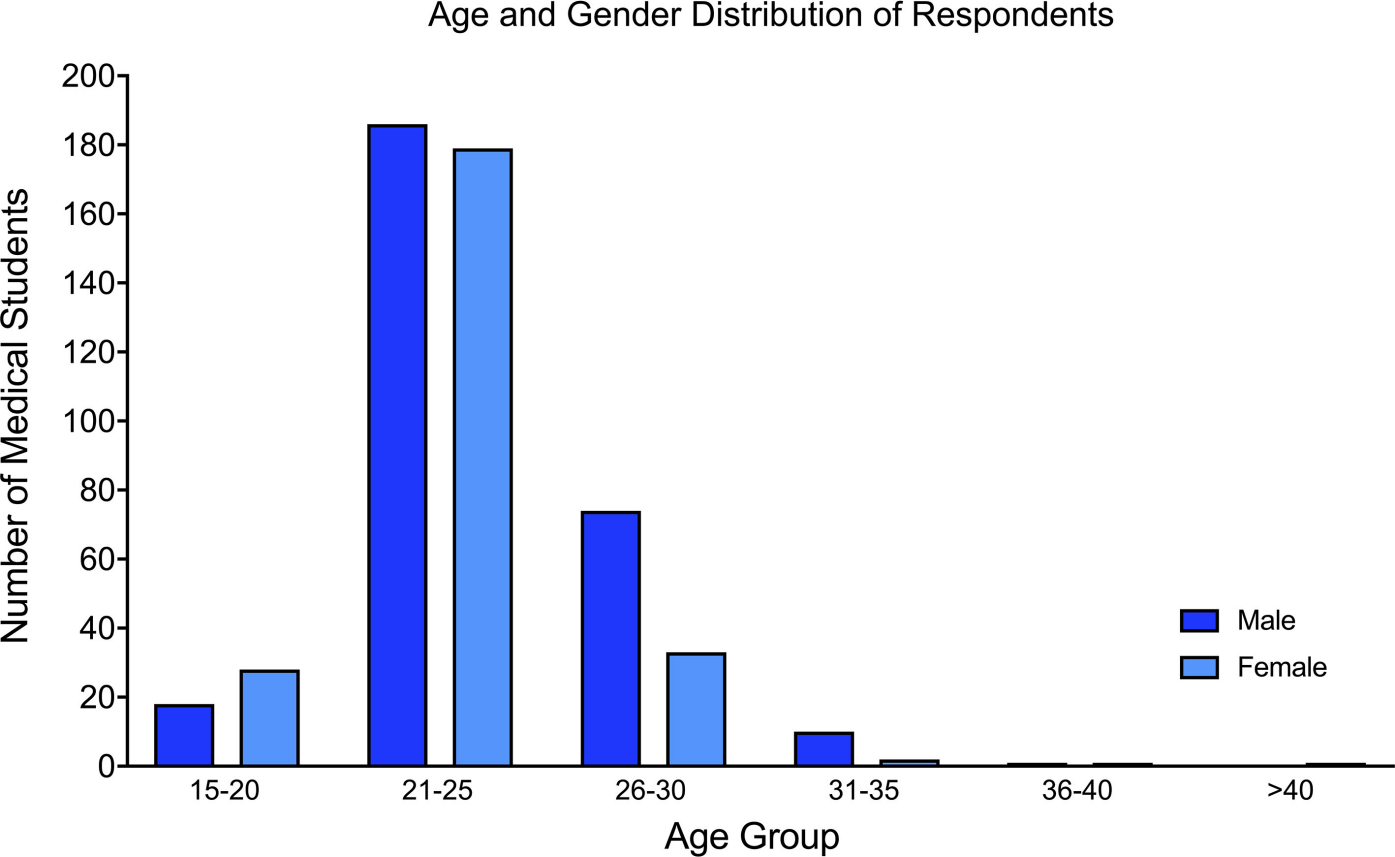
Bar chart showing the age and gender distribution of the participants.

**Figure 2:**
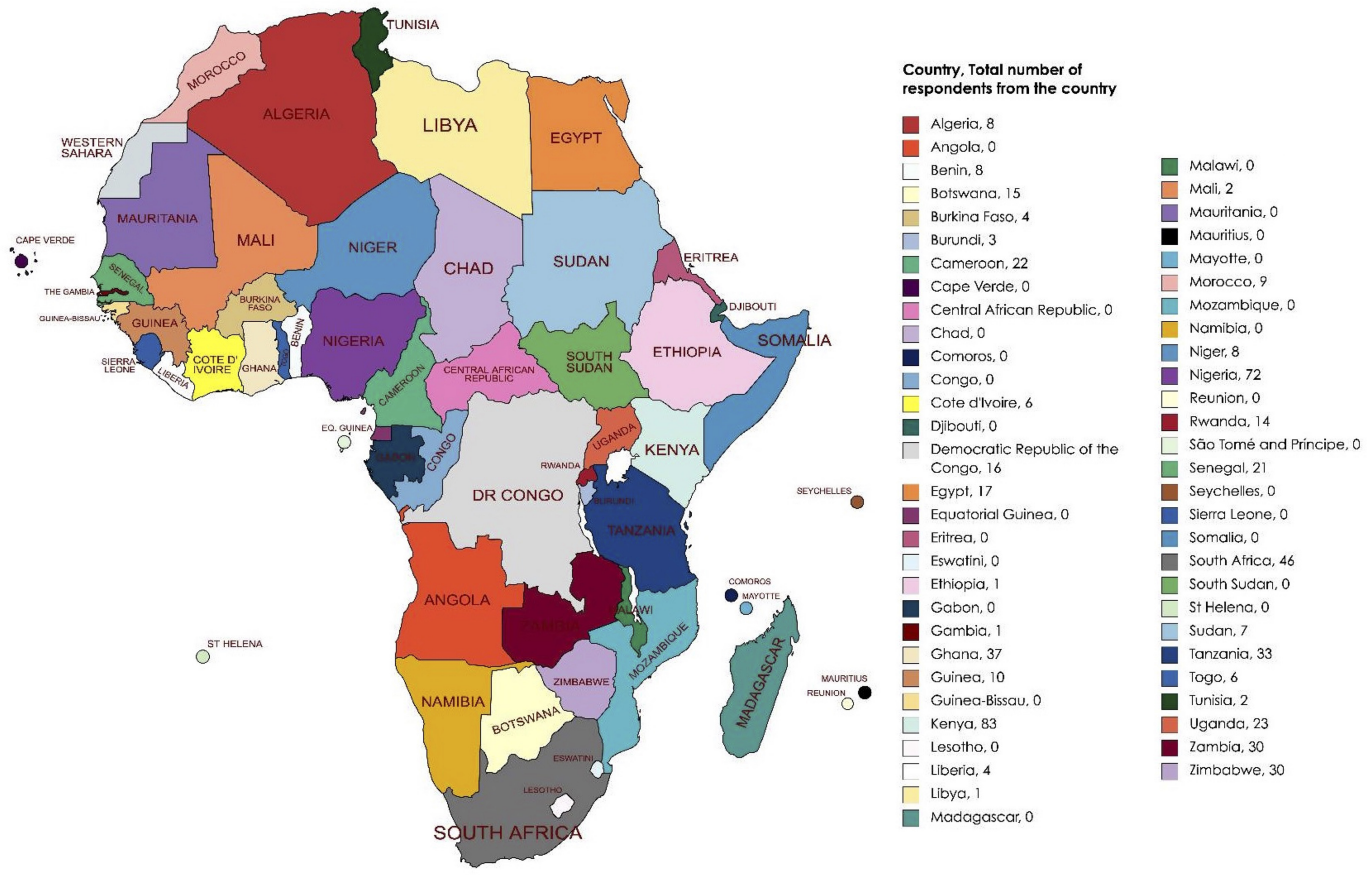
Continental heat map of African countries and number of students that responded to the survey.

Data was provided from 539 medical students from 30 African countries, which represents 55.6% (*n* = 30/54) of all African countries. The majority of respondents were aged between 21 and 25 years old (*n* = 370/539, 68.6%; Figure 1) and a slight majority were male (*n* = 289/539, 53.6%; Table 1). Of the 30 African countries surveyed, the country with the highest number of respondents was Kenya (*n* = 83/539, 15.4%), followed by Nigeria (*n* = 72/539, 13.4%), and South Africa (*n* = 46/539, 8.6%; Figure 2). Most students (504/539, 93.5%) self-reported that they were from medical schools located in urban areas, while the rest stated that their medical schools were in rural areas (Table 1).

**Table 1:** Sociodemographic characteristics of surveyed African medical students.

**Sociodemographic characteristics**	**Number of respondents, *n* (%)**
**Total number of participants**	***N* = 539**
Age
15–20 years	46 (8.5%)
21–25 years	370 (68.6%)
26–30 years	108 (20.0%)
31–35 years	12 (2.2%)
36–40 years	2 (0.4%)
>40 years	1 (0.2%)
Gender
Male	289 (53.6%)
Female	244 (45.3%)
Non-binary	1 (0.2%)
Prefer not to say	5 (0.9%)
Geographical location of institution/hospital
Rural	35 (6.5%)
Urban	504 (93.5%)

No data was missing even though questions 7, 8, and 9 in the questionnaire was dependent on the response to question 6 (Appendix 1).

### Medical Students' Exposure to Neurosurgery

**Figure 3:**
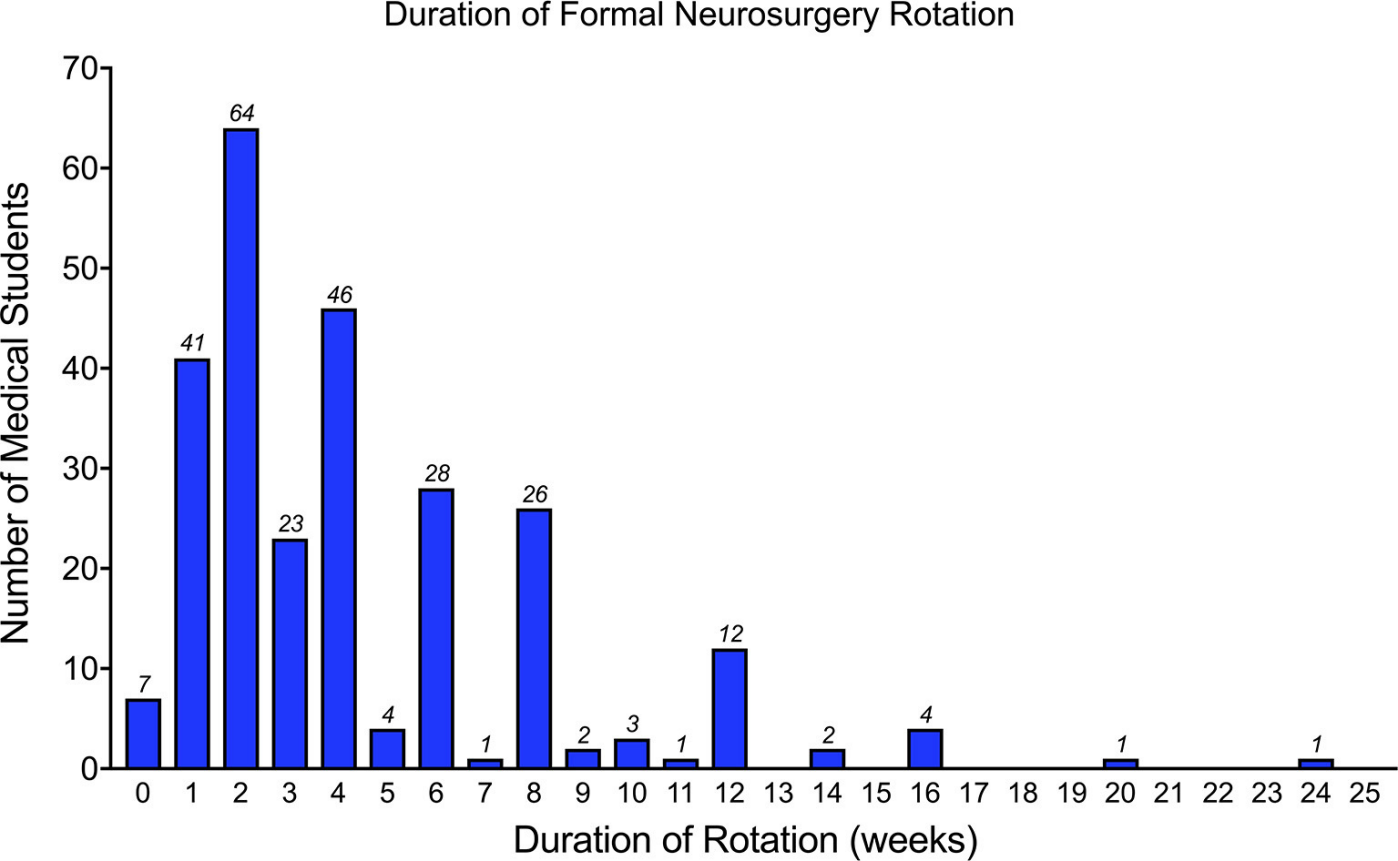
Bar chart showing the duration of clinical neurosurgical rotation, in weeks, of the respondents.

Importantly, 312 (57.8%) students reported a lack of a neurosurgery training program at their base institution (Table 2). A total of 278 (51.6%) medical students had participated in a formal neurosurgery rotation. Of these 278 students, the majority of students had participated in rotations lasting 4 weeks or less (*n* = 181/278, 65.1%), while 33 students (11.8%) had between 4 and 8 (defined as more than four but less than eight) weeks experience, and 52 students (18.2%) had 8 weeks or longer experience on neurosurgical rotation (Figure 3). Twelve students (4.3%) did not report the duration length of their rotation. Only 113 (40.6%) students had a dedicated neurosurgery rotation, while 165 (59.4%) reported a mixed rotation with other specialties.

**Table 2:** Characteristics of neurosurgery experience among African medical students.

**Neurosurgery experience characteristics**	**Number of respondents, *n* (%)**
**Total number of participants**	***N* = 539**
Formal neurosurgery rotation	278 (51.6%)
Exposure to inpatient care	227 (42.1%)
Exposure to outpatient care	153 (28.4%)
Elective surgery	146 (27.1%)
Emergency surgery	92 (17.1%)
Academic meetings	131 (24.3%)
Morbidity and mortality meetings	45 (8.3%)
Ward rounds	201 (37.3%)
Lectures	179 (33.2%)
Bedside tutorials	174 (32.3%)
Informal neurosurgery exposure	160 (29.7%)
Webinar	68 (12.6%)
Research	63 (11.7%)
Workshop	36 (6.7%)
Conference	36 (6.7%)
Elective	15 (2.8%)
Other	4 (0.7%)
Neurosurgery experience outside Africa	15 (2.8%)
Presence of home institution's neurosurgery program	
Yes	227 (42.1%)
No	175 (32.5%)
Unsure	137 (25.4%)

Among 278 students, 227 (81.7%) of students were exposed to inpatient care management, while 153 (55.0%) had participated in outpatient care. In addition, 179 (64.4%) students received teaching through neurosurgery lectures (*n* = 179/278, 64.4%), and 174 (62.5%) had bedside tutorials for neurosurgery (Table 2). In terms of operating room exposure, a more significant proportion of students reported observing or participating in elective neurosurgery cases (*n* = 146/278; 52.5%) than emergency cases (*n* = 92/278; 33.1%). Only 15 (5.4%) students also had exposure to neurosurgery outside of Africa (Table 2). When asked to compare their experiences abroad against those in their home countries, the general consensus among students was the differences between the quality of neurosurgical care delivered. This includes “access to better equipment” and “more neurosurgeons in the workforce” abroad.

Among all 539 students, 160 (29.7%) students reported to have had informal neurosurgery exposure (Table 2). The most common form of informal exposure to neurosurgery were through webinars (*n* = 68/539, 12.6%), followed by research (*n* = 63/539, 11.7%), workshops (*n* = 36/539, 6.7%), and conferences (6.7%).

### Perception of Neurosurgery

Some perceptions of neurosurgery differed between medical students with and without formal neurosurgery rotation experience (Table 3). Of note, students with neurosurgery rotation experience reported a stronger agreement that it is more difficult for women to pursue a career in neurosurgery than those with no neurosurgery rotation experience (*p* = 0.0001). Students with neurosurgery rotation experience also showed stronger agreement that the clinical experience influenced their perception of a neurosurgical career, when compared to non-rotators (*p* = 0.03). Perception of neurosurgery training as emotionally draining was significantly different between the two groups (*p* = 0.001). Students with neurosurgery rotation experience also reported a stronger agreement with the perception of pleasant personalities in the field of neurosurgery, than non-rotators (*p* = 0.04). Interestingly, students with previous formal clinical rotation experience expressed a lower likelihood of pursuing a career in neurosurgery than those without (*p* = 0.02).

**Table 3:** Perception of neurosurgery by formal neurosurgery clinical rotation experience.

**Perception of neurosurgery (1 = strongly disagree, 5 = strongly agree)**	**Rotators**		**Non-rotators**		***p*-value**
**Number of participants**	***N*** **=** **278**		***N*** **=** **261**		
	**Mean (SD)**	**Median (IQR)^a^**	**Mean (SD)**	**Median (IQR)^a^**	
The range of operations performed by neurosurgeons is highly diverse	3.69 (1.09)	4 (3–5)	3.84 (1.15)	4 (3–5)	0.08
The future of neurosurgery is bright	4.04 (1.04)	4 (3–5)	4.01 (1.12)	4 (3–5)	0.98
Neurosurgery is emotionally draining for residents and attendings	3.97 (1.13)	4 (3–5)	3.70 (1.07)	4 (3–5)	0.001^*^
Neurosurgery residency training is very difficult	3.93 (1.08)	4 (3–5)	3.80 (1.10)	4 (3–5)	0.14
Neurosurgeons are financially secured	3.58 (1.16)	4 (3–5)	3.69 (1.13)	4 (3–5)	0.34
Becoming a neurosurgeon and having a family is achievable	3.65 (1.09)	4 (3–5)	3.70 (1.15)	4 (3–5)	0.46
It is more difficult for women to pursue a career in neurosurgery	3.46 (1.29)	4 (3–5)	2.99 (1.43)	3 (2–4)	0.0001^*^
I agree that exposure to neurosurgery (or lack thereof) has influenced your perception about a neurosurgical career	4.08 (1.06)	4 (4–5)	3.79 (1.31)	4 (3–5)	0.03^*^
You are likely to pursue a career in neurosurgery	3.03 (1.45)	3 (2–4)	3.32 (1.34)	3 (3–5)	0.02^*^
The outcome of neurosurgical patients is excellent	3.01 (1.02)		3.07 (1.01)		0.47^b^
In the field of neurosurgery, the personalities of attendings and collegiality between faculty is very pleasant and collegial	3.37 (1.05)		3.18 (0.97)		0.04^*^,^b^
Neurosurgeons have a high quality of life	3.13 (1.04)		3.15 (1.00)		0.79,^b^

a *Median and interquartile range (IQR) are reported and p-value were obtained from Wilcoxon rank-sum test due to non-normality*.

b *Obtained from two-sample t-test*.

* *p-value < 0.05*.

### Interest in a Neurosurgical Career

Among 278 medical students with formal neurosurgery experience, 88 (31.7%) expressed definite interest in a neurosurgical career, while 190 (68.3%) reported no or possible interest. Of 261 medical students without formal rotation experience, 109 (41.8%) reported clear interest in pursuing neurosurgery, and 152 (58.2%) declared no or possible interests.

Considering students attending medical schools in perceived urban settings, the odds of having an interest in a career in neurosurgery were 60% lower in rotators in comparison to non-rotators (OR = 0.40, 95% CI = 0.25–0.64, *p* < 0.001; Table 4) after adjusting for gender. In contrast, this adjusted association was not observed in medical students who attend medical schools in rural areas (OR = 2.28, 95% CI = 0.51–10.18, *p* = 0.278). This analysis is, however, potentially limited by the relatively low number of medical students situated in rural areas (35, 6.5%). Additionally, the interaction between geographical location and formal neurosurgery rotation experience was statistically significant (*p* = 0.02). Importantly, students who had more than 4 weeks of neurosurgical exposure were 1.75 times more likely to express a career interest in neurosurgery than those without (OR = 1.75, 95% CI = 1.02–3.01, *p* < 0.04) when previous rotation experience, gender, and urban/rural location were controlled. Men were 3.22 times more likely than women to express interest in a neurosurgical career (OR = 3.22, 95% CI = 2.18–4.76, *p* < 0.001) after adjusting for previous formal rotation, neurosurgical experience of more than 4 weeks, and urban/rural location.

**Table 4:** Neurosurgical career interest based on adjusted logistic regression model.

**Factors**	**OR**	**95% CI**	**Adjusted *p*-value**
Urban medical students with formal neurosurgery rotation vs. without	0.40	0.25–0.64	<0.001^*^
Rural medical students with formal neurosurgery rotation vs. without	2.28	0.51–10.18	0.278
Formal neurosurgical exposure for more than 4 weeks vs. 4 weeks or less	1.75	1.02–3.01	0.04^*^
Male vs. female	3.22	2.18–4.76	<0.001^*^

* *p-value < 0.05*.

## Discussion

### Summary of Findings

In this study, we assess the perception and interest of clinical medical students in Africa toward a career in neurosurgery. The majority of participants were within the 21–25-year-old age group and there was a slight male preponderance. Regarding neurosurgery exposure, more than half of the students surveyed have had experience of a formal clinical rotation in neurosurgery. Of the students with neurosurgery rotation experience, the majority had a rotation lasting 4 weeks or less. Inpatient care was the most commonly reported form of teaching in a clinical rotation. In terms of operating room exposure, more students had participated or observed elective neurosurgical cases as opposed to emergency cases.

The majority of students are of the perception that it is more difficult for women to pursue a career in neurosurgery, compared to men, and that neurosurgical training is emotionally draining. Indeed, this may, at least partially, explain why collectively students with experience of a formal clinical rotation in neurosurgery of all durations are less likely to demonstrate an interest in pursuing a career in neurosurgery. Students who have gained both local and international perspectives in the field of neurosurgery highlight the under-resourced nature of the specialty in Africa. This provides insight into potentially concerning findings regarding the perception of neurosurgery amongst medical students in Africa. Nevertheless, our finding that students with >4 weeks formal exposure to neurosurgery, either during dedicated or mixed speciality placements, are more likely to express a career interest in neurosurgery highlights an opportunity for future evidence-based curriculum modifications to help inspire the next generation of neurosurgeons and begin tackling the continental workforce deficit.

### Implications

The presence of a neurosurgical rotation or the lack thereof can inevitably impact students' decisions on whether they should pursue a career in neurosurgery. In our survey, respondents who had a formal rotation in neurosurgery agreed that the experience influenced their perception of a neurosurgical career. This is expected when one has been exposed to a clinical specialty. The clinical experience exposes students to the intimacies of this field, allowing students to either nurture a willingness to pursue a career in this field or dismiss the profession on an informed basis. Conversely, students who lack prior exposure may consider pursuit of a neurosurgical career based predominantly on societal views or presumed prestige, whilst failing to appreciate potential sacrifices common in the profession. In a survey of third-year, fourth-year, and fifth-year medical students from the Royal College of Surgeons in Ireland on their perception of a neurosurgical career, 92% acknowledged high prestige and income attached to neurosurgery as positives (8). However, the pitfalls of a career in neurosurgery included 98% of respondents feeling that the training was too long, 97% feeling that the operations were too long, and 87% feeling that a neurosurgical career impeded on the quality of a family life (8). Although our study did not specifically examine the perception of prestige, most students responding to our survey agreed that neurosurgeons are financially secure and that the future of neurosurgery is bright. This can be related to a study conducted among 256 Nigerian final-year medical students at a single institution. Balogun et al. reported in the study that perception of prestige and high-income potential was associated with increased interest toward a neurosurgical career (9). Akhigbe et al., also reported that the neurosurgery's high-income and perceived prestige were driving factors for medical students' interest in neurosurgery (8). These results highlight the importance of a neurosurgical clinical rotation in medical school, to ensure that any aspiring neurosurgeon is provided with the opportunity to gain a clear, truthful appreciation of the specialty before making a decision on which specialty to pursue. The lack of medical student exposure to neurosurgery is likely contributed to by many factors. However, only 42.1% of students we surveyed were able to confirm the presence of a neurosurgical programme at their base institution highlighting a fundamental lack of opportunity. This suggests that many medical institutions may need to partner with external neurosurgical centres to provide their students with opportunities for exposure to this speciality.

A key finding in our study was that students with formal neurosurgical exposure had a stronger consensus, than those without, that it is more difficult for women to pursue neurosurgery as a career. Such views may be influenced by students having witnessed, first-hand, the lack of female representation in neurosurgical departments at almost all levels, from the head of departments to trainees. This creates a void of potential mentors, guidance, and role models for aspiring female neurosurgeons and perpetuates the barriers to women considering a career in neurosurgery (10). Although there are increasing numbers of female neurosurgeons and trainees globally, stark gender inequalities still exist and this is even more apparent in developing countries (11). Throughout Africa, the issue of the lack of female representation in neurosurgery is attributed to many deep-seated factors, such as the inequality of access to education for women. In recent years, work has begun to educate and empower women who wish to pursue a career in the health sector, and specifically, surgical fields. However, the issue within neurosurgery persists as there is a paucity of representation of current female neurosurgeons in academia and leadership positions (12). Notwithstanding, it is important to recognize that the issue goes beyond providing female neurosurgeons equal opportunities. Currently, there are no potential training arrangements for neurosurgeons to undertake less than full-time training (LTFT)-a policy implemented in countries such as the United Kingdom (13). Given the demanding nature of neurosurgery, the lack of LTFT opportunities may be a barrier to perceived feasibility of pursuing the field, especially if one wishes to allocate more time for family whilst continuing their progression in training. Moreover, there is also no easily accessible data on the impact of maternity leave on a neurosurgeon's salary. The uncertainty that comes with the lack of transparency and/or lack of attention given to discussions about remuneration during maternity leave could also be a potential barrier to women pursuing neurosurgery. A reform of local policies by national governments and stakeholders addressing these issues would be a critical step to improve the perception of neurosurgery to women and to level the playing field for them with the flexibility they may need during their training. Furthermore, a potential focus for future research would include detailed evaluation of the attitudes and perceptions of aspiring female neurosurgeons in Africa toward pursuing a career in the specialty. This would be crucial in gaining in-depth understanding of the relevant perceived barriers, and thus, providing valuable data to the literature that may be useful in influencing change to neurosurgery programs in Africa.

Perception of neurosurgical training as emotionally draining was significantly different between those who had a rotation and those who did not. It is well known that neurosurgical training is time-consuming and demanding, and when students have had some exposure to this, their perceptions may become more grounded and realistic. The perception that neurosurgery is emotionally draining may be associated with a perceived lack of work-life balance, which is a vital factor deterring medical students from a career in neurosurgery consistent with previous surveys completed in the West (14, 15). For students who have not had a formal rotation, the demands of the career can be underestimated or glamorized by the media and perceived prestige.

Another factor influencing the diminished interest of students with neurosurgery rotation experience compared to students without neurosurgery rotation experience may include the reduced surgical and practical nature of the rotation. In a study of 76 students, Burford et al., reported that the most influential factor in attracting medical students to neurosurgical careers was the surgical approaches (14). Of the 278 students who were able to complete a formal neurosurgical rotation, only 44.2% reported having any surgical exposure. This low exposure to practical neurosurgery could contribute to diminished interest. Improving the practical aspect of these rotations could foster more interest and support budding African neurosurgeons.

A positive finding of our study was that respondents who had a neurosurgical rotation reported higher agreement that pleasant personalities and collegiality within the field of neurosurgery were prevalent. This demonstrates the positive impact rotations could have on dismantling the societally imposed negative stereotypes that have plagued neurosurgery. It is often assumed that the neurosurgical specialty is impossibly challenging, devoid of life, and can damage family life (8). These sentiments keep away potentially strong candidates, and without personable exposure, these inaccurate stereotypes may otherwise be held as universal truths. The results of the study observed are encouraging as an increased perception of pleasant personalities and collegiality within the field may facilitate students interested in entering the field to reach out and seek guidance and mentorship. This is key as strong mentorship is associated with a more successful matching of medical students into neurosurgical residency programs (16). Mentorship is also a significant factor influencing sub-specialty choice among medical students in general (17). This perception may also influence the pursuit of academic interests, collaboration, and career opportunities which are significant factors influencing specialty choice among medical students (17).

Overall, in contrast to our central hypothesis, students with formal clinical rotation experience of any length in neurosurgery were less likely to pursue a career in neurosurgery than those with no formal neurosurgical experience. This might reflect more students with a predetermined interest in other career paths in larger medical schools where students are more likely to gain exposure to a broader range of specialties, in addition to neurosurgery. This also highlights how career path selection amongst medical students is multifactorial and interdependent between the various specialities available (16).

Interestingly, our analysis demonstrated that students with more than 4 weeks of experience in a formal neurosurgical rotation were 1.75 times more likely to pursue a career in neurosurgery than those with exposure lasting 4 weeks or less. This highlights the importance of lengthened exposure to this field to medical students as the added time allows students to gain greater experience within the specialty and to seek opportunities that may maintain their interest in the long term. These opportunities could be in the form of establishing mentorship, conducting a research project with the local department and observing/assisting in more neurosurgical cases. Similar to our work, Balogun et al. observed only 64.5% of students on clinical rotations felt they received adequate neurosurgical teaching, and 47% of students felt they did not spend enough valuable time with the neurosurgical consultants (9). This may, at least in part, explain our findings that a shorter clinical rotation is associated with decreased interest in neurosurgery. Although it is possible that some students with a strong pre-existing interest in neurosurgery may have been able to self-arrange longer formal rotations in neurosurgery, it is unlikely to have been common since internal student selected components are not available within the curricula of many medical schools across Africa. Therefore, it is imperative that medical schools in Africa review their curricula and consider lengthening exposure to neurosurgical rotations as appropriate, even if this is within the context of mixed rotations. Stakeholders and national organizations could also play a part in offering bursaries for African medical students to undertake neurosurgical clinical electives during term time and/or holiday periods as a means to provide students with the opportunity to expand and consolidate their knowledge and skills and to promote continuity in their learning in the specialty.

## Challenges and Limitations

Although our study analyses the responses of 539 medical students across 30 nations within Africa, there was a preponderance of responses from students based in Kenya, Nigeria, and South Africa. Therefore, our sample distribution and size may reflect a degree of underrepresentation of clinical medical students within some nations across Africa. Additionally, the survey was only provided in English or French. Distribution of the survey in other languages, such as Portuguese and Arabic, might have helped enhance the diversity of representation and reach a larger population of students. However, due to resource constraints, this was not possible. Also, given the mode of data collection for the survey, some preclinical (ineligible) medical students may have responded. Additionally, students in rural areas or areas with an unstable internet connection or poor access to the internet may have been disproportionately unable to participate in the survey.

## Recommendations

The expansion of neurosurgical training is required to tackle the current workforce deficit throughout the continent of Africa and provide safe, timely neurosurgical care. The deficit in neurosurgical training, education, and exposure is contributed to by a lack of resources, infrastructure, and governance within the medical school curriculum. Following the results of our study, we have compiled a list of recommendations that can be implemented to increase interest in neurosurgery within Africa.

The emphasis on increasing diversity and inclusion within the neurosurgical field was strongly suggested throughout our data collection. The lack of female representation within the neurosurgical field has been a great detriment toward the progression of inclusivity and the expansion of neurosurgical availability in general. To help overcome this hurdle, we recommend increasing the number of students with a formal rotation over 4 weeks duration in clinical neurosurgery, in addition to increasing the number of female students rotating within these programs. Where feasible, resources to provide rotation-specific mentorship could be prioritized toward female medical students and, ideally, delivered by female trainees or consultants-even if greater implementation of virtual/distance mentorship programs is required to ensure this is achievable. Similar to the beneficial effects of neurosurgery interest groups throughout the global West including the UK (18) and US (19), the establishment and promotion of neurosurgery interest groups within and between medical schools throughout Africa could help enhance more equitable neurosurgical exposure and opportunities for mentorship. Initiatives such as the Association of Future African Neurosurgeons (AFAN) may play a key role in this respect and should be supported (20).

In addition to increasing diversity within neurosurgery rotations, we propose offering a dedicated neurosurgical rotation that does not intertwine with other surgical specialties. Often, neurosurgical rotations are intertwined with other surgical rotations such as obstetrics and gynecology, general surgery, and orthopedics (21). The lack of dedicated time within the neurosurgical field fails to allow for the complete acclimation into the neurosurgery specialty and may reduce student's ability to gain broader appreciation of what is required of a neurosurgeon during training. Therefore, having a dedicated neurosurgical rotation could potentially increase neurosurgical interest and help debunk any myths surrounding the neurosurgical specialty, allowing students to make well-informed decisions about future specialty choices. Nevertheless, our data suggests that even mixed speciality placements which incorporate neurosurgical exposure are still likely to be beneficial in nurturing career interest in neurosurgery, so long as these rotations last >4 weeks.

Collaboration is recommended between institutions within Africa and larger institutions in the global West as the sharing of education resources, funding, and training opportunities will increase the number of students that show interest in the neurosurgical field. For example, partnerships include the Duke Global Neurosurgery and Neurosciences (DGNN) working at Mulago Hospital in Uganda and organizations such as Global Partners in Anesthesia and Surgery (GPAS), which are great examples of collaborations that have helped continue to broaden, improve, and diversify the field of neurosurgery within Africa (22).

## Conclusion

Neurosurgical exposure is a vital determinant in the perception and interest of African medical students toward pursuing a career in neurosurgery. Our study has identified the most relevant factors responsible for interest or lack of such interest in a neurosurgical career. A large majority of African medical students do not have optimal exposure to neurosurgical rotations. Of those students who have neurosurgical exposure, there is often an inadequacy in the inpatient and outpatient exposure during rotations. The perceived gender inequality in most neurosurgical centers in Africa is also a deterring factor as females in neurosurgery are exquisitely underrepresented, and this may discourage female medical students from pursuing neurosurgery as a career. These findings and our recommendations can help inform more standardized and equitable access to neurosurgical rotations in medical training centers across Africa, with greater gender inclusion in advocacy, education, and policy development for neurosurgical training in Africa.

## Data Availability Statement

The raw data supporting the conclusions of this article will be made available by the authors, without undue reservation.

## Ethics Statement

Ethical review and approval was not required for the study on human participants in accordance with the local legislation and institutional requirements. The patients/participants provided their written informed consent to participate in this study.

## Author Contributions

OD: conceptualization, data analysis, writing, editing, and critical revisions of the manuscript. SO: data analysis, data visualization, writing, editing, and critical revisions of the manuscript. GB, YK, EO, EA, KN, AA, OO, LK, AO, UB, MB, and DO: writing, editing, and critical revisions of the manuscript. CL: writing, data analysis, editing, and critical revisions of the manuscript. OR: conceptualization, writing, editing, and critical revisions of the manuscript.

## Funding

OR is supported by an NIHR clinical lectureship in neurosurgery.

## Conflict of Interest

The authors declare that the research was conducted in the absence of any commercial or financial relationships that could be construed as a potential conflict of interest.

## Publisher's Note

All claims expressed in this article are solely those of the authors and do not necessarily represent those of their affiliated organizations, or those of the publisher, the editors and the reviewers. Any product that may be evaluated in this article, or claim that may be made by its manufacturer, is not guaranteed or endorsed by the publisher.
