# Supplementary material for: Evaluating the Impact of Neurosurgical Rotation Experience in Africa on the Interest and Perception of Medical Students Towards a Career in Neurosurgery: A Continental, Multi-Centre, Cross-Sectional Study
Source: Front Surg. 2022 Feb 10;9:766325. doi: 10.3389/fsurg.2022.766325 (PMC8866575; doi:10.3389/fsurg.2022.766325)
Supplement: Supplementary file 1 [file Table_1.pdf]

Appendix

Appendix 1. This table shows the list of questions included in the 27-item questionnaire distributed to the medical students.

**INTEREST AND PERCEPTION OF CLINICAL MEDICAL STUDENT TOWARDS A NEUROSURGICAL CAREER**<br><br>This survey is only meant for clinical medical students in African universities.<br>This survey will help us to ascertain the impact of a neurosurgery rotation and lack of neurosurgery rotation on the perception of clinical students towards a neurosurgical career.<br>Data collected from this survey is non-identifiable and will be used for research purposes.<br><br>** Required*
**Question 1**<br>By filling this form, you consent that you understand and agree with terms stated above. *<br>● Yes<br>● No<br>*(Multiple choice)*
**Section A**: Sociodemographic background of respondents
**Question 2**<br>Gender *<br>● Female<br>● Male<br>● Non-binary<br>● Prefer not to say<br>*(Multiple choice)*
**Question 3**<br>Age (years) *<br>● 15-20<br>● 21-25<br>● 26-30<br>● 31-35<br>● 36-40<br>● >40<br>*(Multiple choice)*
**Question 4**<br>Country of study *<br>● Algeria<br>● Angola

- Benin Republic
- Botswana
- Burkina Faso
- Burundi
- Cabo Verde
- Cameroon
- Central African Republic (CAR)
- Chad
- Comoros
- Congo
- "Democratic Republic of the Congo"
- "Republic of the Côte d'Ivoire"
- Djibouti
- Egypt
- Equatorial Guinea
- Eritrea
- Eswatini(Swaziland)
- Ethiopia
- Gabon
- Gambia
- Ghana
- Guinea-Bissau
- Guinea Conakry
- Kenya
- Lesotho
- Liberia
- Libya
- Madagascar
- Malawi
- Mali
- Mauritania
- Mauritius
- Morocco
- Mozambique
- Namibia
- Niger
- Nigeria
- Rwanda
- Sao Tome and Principe Senegal
- Seychelles
- Sierra Leone
- Somalia
- South Africa
- South Sudan
- Sudan
- Tanzania

This is a provisional file, not the final typeset article

- Togo
- Tunisia
- Uganda
- Zambia
- Zimbabwe

(Multiple choice)

Question 5
Geographical location of institution/hospital *

- Urban
- Rural

(Multiple choice)

Section B: Neurosurgery exposure of respondents

Question 6
Have you been exposed to neurosurgery in a formalised clinical rotation? *

- Yes
- No

(Multiple choice)

Question 7
If you answered yes to the previous question, what was the length of this rotation? (weeks)
(Free text)

Question 8
What was the nature of this rotation?

- Dedicated neurosurgery rotation
- Mixed rotation with other specialties

(Multiple choice)

Question 9
In these rotations, what were you exposed to (either as a participant or observer)?
Check all that apply.

- Exposure to patients managed as inpatients
- Exposure to patients managed as outpatients
- Elective surgery
- Emergency surgery
- Academic meetings
- Morbidity and mortality meetings
- Ward rounds
- Lectures
- Bedside tutorials
- Other: *(free text)*

(Checkboxes)

Question 10
Have you had any other neurosurgery experience outside of a formal clinical rotation? *

Check all that apply.<br>● Neurosurgery research<br>● Neurosurgery webinar<br>● Neurosurgery workshop<br>● Neurosurgery conference<br>● Neurosurgery elective<br>● None<br>● Other: *(free text)*<br>*(Checkboxes)*
**Question 11**<br>Does your school have a neurosurgery training program? *<br>● Yes<br>● No<br>● I don't know<br>*(Multiple choice)*
**Question 12**<br>Have you had any exposure to neurosurgery outside Africa? *<br>● Yes<br>● No<br>*(Multiple choice)*
**Question 13**<br>If yes, what is your take on the quality of neurosurgery in your home country and the country you visited?<br>*(Free text)*
**Section C**: Perception towards a neurosurgical career
**Question 14**<br>The range of operations performed by neurosurgeons is highly diverse<br>● Strongly agree<br>● Agree<br>● Neutral<br>● Disagree<br>● Strongly disagree<br>*(Likert scale)*
**Question 15**<br>The future of neurosurgery is bright *<br>● Strongly agree<br>● Agree<br>● Neutral<br>● Disagree<br>● Strongly disagree<br>*(Likert scale)*

This is a provisional file, not the final typeset article

Question 16
The outcome of neurosurgical patients is excellent *
- Strongly agree
- Agree
- Neutral
- Disagree
- Strongly disagree

(Likert scale)

Question 17
Neurosurgery is very emotionally draining for residents and attendings *
- Strongly agree
- Agree
- Neutral
- Disagree
- Strongly disagree

(Likert scale)

Question 18
Neurosurgery residency training is very difficult *
- Strongly agree
- Agree
- Neutral
- Disagree
- Strongly disagree

(Likert scale)

Question 19
In the field of neurosurgery, the personalities of attendings and collegiality between faculty is very pleasant and collegial *
- Strongly agree
- Agree
- Neutral
- Disagree
- Strongly disagree

(Likert scale)

Question 20
Neurosurgeons are financially secure *
- Strongly agree
- Agree
- Neutral
- Disagree
- Strongly disagree

(Likert scale)

Question 21

Neurosurgeons have a good quality of life *
- Strongly agree
- Agree
- Neutral
- Disagree
- Strongly disagree

(Likert scale)

Question 22
Becoming a neurosurgeon and having a family is achievable
- Strongly agree
- Agree
- Neutral
- Disagree
- Strongly disagree

(Likert scale)

Question 23
It is more difficult for women to pursue a career in neurosurgery *
- Strongly agree
- Agree
- Neutral
- Disagree
- Strongly disagree

(Likert scale)

Question 24
Do you agree that exposure to neurosurgery (or lack thereof) has influenced your perception about a neurosurgical career? *
- Strongly agree
- Agree
- Neutral
- Disagree
- Strongly disagree

(Likert scale)

Section D: Interest in a neurosurgical career

Question 25
Do you want to pursue a career in neurosurgery? *
- Yes
- No
- Maybe

(Multiple choice)

Question 26
You are likely to pursue a career in neurosurgery *
- Strongly agree

This is a provisional file, not the final typeset article

● Agree<br>● Neutral<br>● Disagree<br>● Strongly disagree<br>*(Likert scale)*
**Question 27**<br>Would you consider a neurosurgery elective? *<br>● Yes<br>● No<br>*(Multiple choice)*
